# A multicentre neonatal interventional randomised controlled trial of nebulized surfactant for preterm infants with respiratory distress: Neo-INSPIRe trial protocol

**DOI:** 10.1186/s12887-023-04296-4

**Published:** 2023-09-19

**Authors:** Ilse Lategan, David Durand, Michael Harrison, Firdose Nakwa, Lizelle Van Wyk, Sithembiso Velaphi, Alan Horn, Gugu Kali, Roger Soll, Danielle Ehret, Heather Zar, Lloyd Tooke

**Affiliations:** 1 Department of Paediatrics and Child Health, University of Cape Town, Cape Town, South Africa; 2 Aerogen Pharma, San Mateo, CA USA; 3 Groote Schuur Hospital Neonatal Unit, Neonatal Department, Groote Schuur Hospital, Old Main Building, Cape Town, South Africa; 4 Department of Paediatrics and Child Health, University of the Witwatersrand, Johannesburg, South Africa; 5 Chris Hani Baragwanath Hospital Neonatal Unit, Johannesburg, South Africa; 6 Department of Paediatrics and Child Health, Stellenbosch University, Cape Town, South Africa; 7 Tygerberg Hospital Neonatal Unit, Cape Town, South Africa; 8 Vermont Oxford Network, Burlington, VT USA; 9 University of Vermont Larner College of Medicine, Pediatrics, Burlington, VT USA; 10 Unit on Child and Adolescent Health, South African Medical Research Council, Cape Town, South Africa

**Keywords:** Surfactant, Aerosolized, Nebulized, Preterm, Neonatal, RDS

## Abstract

**Introduction:**

Respiratory distress syndrome in preterm infants is an important cause of morbidity and mortality. Less invasive methods of surfactant administration, along with the use of continuous positive airway pressure (CPAP), have improved outcomes of preterm infants. Aerosolized surfactant can be given without the need for airway instrumentation and may be employed in areas where these skills are scarce. Recent trials from high-resourced countries utilising aerosolized surfactant have had a low quality of evidence and varying outcomes.

**Methods and analysis:**

The Neo-INSPIRe trial is an unblinded, multicentre, randomised trial of a novel aerosolized surfactant drug/device combination. Inclusion criteria include preterm infants of 27–34^+6^ weeks’ gestational age who weigh 900-1999g and who require CPAP with a fraction of inspired oxygen (FiO_2_) of 0.25–0.35 in the first 2–24 h of age. Infants are randomised 1:1 to control (CPAP alone) or intervention (CPAP with aerosolized surfactant). The primary outcome is the need for intratracheal bolus surfactant instillation within 72 h of age. Secondary outcomes include the incidence of reaching failure criteria (persistent FiO2 of > 0.40, severe apnoea or severe work of breathing), the need for and duration of ventilation and respiratory support, bronchopulmonary dysplasia and selected co-morbidities of prematurity. Assuming a 40% relative risk reduction to reduce the proportion of infants requiring intratracheal bolus surfactant from 45 to 27%, the study will aim to enrol 232 infants for the study to have a power of 80% to detect a significant difference with a type 1 error of 0.05.

**Ethics and dissemination:**

Ethical approval has been granted by the relevant human research ethics committees at University of Cape Town (HREC 681/2022), University of the Witwatersrand HREC (221112) and Stellenbosch University (M23/02/004).

**Trial registration:**

PACTR202307490670785

**Supplementary Information:**

The online version contains supplementary material available at 10.1186/s12887-023-04296-4.

## Introduction

### Background and rationale

Respiratory Distress Syndrome (RDS) due to pulmonary surfactant deficiency is an important cause of morbidity and mortality in infants born prematurely [1]. Initial stabilization on continuous positive airway pressure (CPAP) together with early rescue surfactant administration has resulted in reduced rates of bronchopulmonary dysplasia (BPD) and death and has become the standard of care in high income countries [2]. Intratracheal bolus surfactant can be delivered by intubation followed by a period of mechanical ventilation (MV), or via the intubation – surfactant – extubation (INSURE) method which reduces the need for ongoing MV. Newer methods of administering surfactant via thin catheter such as less invasive surfactant administration (LISA) or minimally invasive surfactant therapy (MIST) can be employed for infants breathing spontaneously on CPAP and reduces the need for any positive pressure ventilation.

All methods of intratracheal bolus surfactant administration currently in clinical practice require laryngoscopy and instrumentation of the airway. These procedures require training, supervised practice, and skill. Changes in neonatal practice with the increased utility of non-invasive respiratory support for preterm infants has resulted in limited opportunities for doctors to become proficient at neonatal intubation [3]. A large international registry study of more than 2600 neonatal intubations found that less than 50% of intubations were successful on the first attempt and that severe oxygen desaturations were common. Furthermore, an increased number of intubation attempts was associated with adverse events such as oesophageal or right main bronchus intubation, bradycardia, and traumatic injury [3]. This problem is further complicated in low resourced settings where access to standardised airway guidelines and key airway equipment is limited [4].

Aerosolized surfactant represents the least invasive method of surfactant delivery. This method facilitates surfactant administration to a spontaneously breathing infant on CPAP whilst limiting the potential complications associated with intratracheal bolus surfactant administration. Four recent trials using a variety of surfactant preparations and nebulising devices have endeavoured to compare early aerosolized surfactant plus CPAP to CPAP alone, with the primary outcome being the need for intratracheal bolus surfactant (Table 1). The results from these trials were varied; Dani et al. found no benefit whilst Jardine et al. reported a non-significant trend toward reduced rates of instilled surfactant [5, 6]. Cummings et al. conducted the largest study and reported a reduced need for intratracheal bolus surfactant by up to 50% in the aerosolized group, particularly in infants > 31 weeks [7]. Despite the promising findings, there were methodological concerns raised as failure criteria were poorly defined [8]. Minocchieri et al. also showed a reduced need for intubation in the intervention group for infants > 32 weeks but there were also methodological flaws [9]. A meta-analysis of 9 studies examining the efficacy of aerosolized surfactant to prevent early intubation revealed that overall, the quality of evidence was low due to bias [1]. All the studies, however, showed that aerosolized surfactant was safe and well tolerated [5–7, 9, 10].

**Table 1:** Recent trials on aerosolized surfactant

Study (Author/Year)	Gestational Age	Weight	Age at enrolment	Respiratory support method	Entry FiO_2_ requirement or RSS	Findings
**Minocchieri **[9] Level III neonatal unit in Australia, 2019	29–33 + 6	1560 g (mean)	< 4 h	Bubble CPAP	FiO_2_ 0.22–0.30	Early AS may reduce the need for intubation in the first 72 h of age for infants > 32 weeks
**Cummings **[7] Level III/IV neonatal units in USA, 2019	23–41	590–4800 g	> 1–< 12 h	Several NIV types	Initially FiO_2_ 0.25–0.40, then removed	AS reduced the need for intubation and intratracheal bolus surfactant by 50%, particularly in infants > 31 weeks
**Jardine **[6] Level III neonatal units in Australia, 2021	26–30 + 6	1170 g (mean)	15 min—≤ 2 h	nCPAP	Part 1&2: FiO_2_ < 0.30 Part 2 re-treat: RSS ≥ 2.0	Non-significant trend toward reduced rates of CPAP failure and need for intratracheal bolus surfactant in AS group
**Dani **[5] Level III neonatal units in Europe, 2022	28–32	1416 g (mean)	> 1–< 12 h	nCPAP	FiO_2_ 0.25–0.40	AS did not reduce the need for intratracheal bolus surfactant administration in the first 72 h of age compared with CPAP alone
**Aerogen Pharma Phase IIb** Level III neonatal units in USA & Canada, 2020	26–31 + 6	≤ 2000 g	< 24 h	nCPAP or NIV	RSS 1.4–2.0	Trial underway (clincaltrials.gov trial no: NCT03969992)

*Abbreviations*: *g* grams, *h* hours, *min* minutes, *nCPAP* nasal Continuous Positive Airway Pressure, *NIV* Non-invasive ventilation, *FiO*_*2*_ Fraction of inspired Oxygen, *RSS* Respiratory Severity Score (MAP x FiO_2_), *AS* aerosolized surfactant

Preterm infants with RDS born in many low- and middle-income countries (LMICs) have limited access to doctors who are trained and skilled in laryngoscopy and instillation of intratracheal bolus surfactant [11]. In these low resourced countries aerosolized surfactant may have particular relevance as it potentially reduces the severity of RDS and improves outcomes of preterm infants without the need for specialized training or technical skills [12].

### Trial design

This is a multicentre, phase 2, non-blinded, randomised controlled trial. Infants will be randomised in a 1:1 manner to either the intervention or control group, according to a balanced block design. This protocol is reported following the Standard Protocol Items: Recommendations for Interventional Trials guidelines (SPIRIT) [13]. See Supplementary material for SPIRIT checklist.

### Objectives

The primary objective is to determine if aerosolized surfactant given to preterm infants with RDS on nCPAP, compared with nCPAP alone, reduces the need for intratracheal bolus surfactant instillation in the first 72 h of age.

Secondary objectives are:

- To compare the need for repeat intratracheal bolus surfactant dosing between groups
- To compare the need for mechanical ventilation between groups
- To compare the duration of mechanical ventilation, CPAP, high flow nasal cannula (HFNC) and supplementary oxygen between groups
- To compare the rate of adverse events and select comorbidities of prematurity between groups (see Appendix for definitions, included as Supplementary material)
- To compare the incidence of BPD between groups (see Appendix for definitions, included as Supplementary material)
- To compare the incidence of death at any time between groups
- To compare the incidence of death or BPD between groups

## Methods

### Participants, interventions, and outcomes

#### Study setting

The study will enrol infants at three level 3 South African neonatal units that are each affiliated with an academic institution. An observational study was conducted in Cape Town to describe the respiratory support needs and outcomes of preterm infants admitted to these units, and to inform the use case for this interventional trial [14]. All preterm infants with RDS admitted to these units are preferentially managed with nCPAP and inborn infants ≥ 900 g have access to intratracheal bolus surfactant administration and invasive ventilation options.

#### Eligibility criteria

Inclusion criteria:

- Inborn
- Birth weight of 900–1999 g
- 27–34 weeks gestational age
- 2–24 h of age at time of randomisation
- Persistent fractional inspired oxygen (FiO_2_) of 0.25–0.35 on nCPAP at 5–7 cmH2O to maintain peripheral oxygen saturation of 90–95%. FiO_2_ requirement needs to be sustained for at least 15 min.

**Note**: A best estimate of the gestational age in weeks and days will be recorded for all enrolled infants using the following hierarchy:

1. Early (< 20 weeks gestation) ultrasound
2. Postnatal assessment which will include Ballard score and foot length

Exclusion criteria:

- Administration of inotropes and/or intubation prior to enrolment in the delivery room or Neonatal Intensive Care Unit (NICU)
- 5-min Apgar score ≤ 5
- Prior instillation of intratracheal bolus surfactant
- Pneumothorax that requires needle thoracentesis or insertion of an intercostal chest drain
- Life-threatening congenital anomaly
- Known or suspected chromosomal abnormality
- Known or suspected congenital infection
- Enrolment in another interventional study with competing outcomes

### Interventions

#### Investigational product

AeroFact™ (Aerogen Pharma) is an investigational drug/device combination product consisting of bovine-origin surfactant SF-RI 1 and a vibrating mesh nebulizer with a nasal interface. SF-RI 1 (Bovactant) is currently marketed under the name Alveofact™ (Lyomark Pharma GmbH). The AeroFact™ drug delivery system delivers fine droplets (2-3 µm) of aerosolized surfactant, synchronized with inspiration, to spontaneously breathing infants on nCPAP.

#### Intervention group

**Fig. 1:**
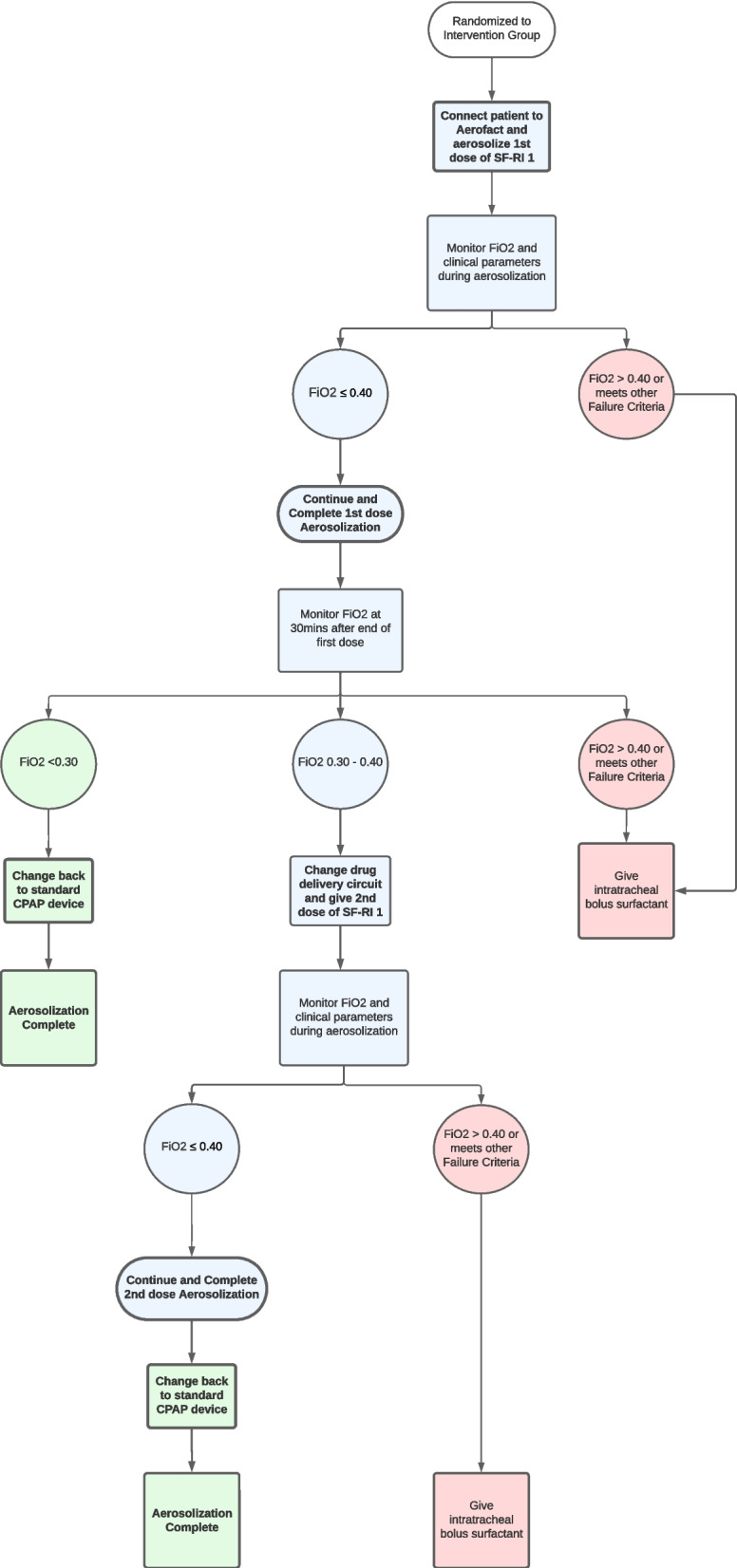
Intervention and redosing flow chart

See Fig. 1 for intervention group flow chart. Following randomisation to the intervention group, infants will receive one dose of aerosolized SF-RI 1 surfactant of 216 mg/kg per dose if they have a persistent FiO_2_ of 0.25–0.35 on nCPAP at 5–7 cmH_2_O to maintain peripheral oxygen saturation of 90–95%. FiO_2_ requirement needs to be sustained for at least 15 min.

Infants may receive one additional dose of aerosolized SF-RI 1 surfactant of 216 mg/kg per dose (for a total of two doses) if 30 min have elapsed since the end of the first dose and they have an ongoing and persistent FiO_2_ of 0.30–0.40 on nCPAP at 5–7 cmH_2_O to maintain peripheral oxygen saturation of 90–95%.

The Infants in the intervention group will be placed on a Fisher & Paykel (F&P™) Bubble CPAP system for the duration of aerosolization as a dual limb inspiratory and expiratory system is required for use with the AeroFact^TM^ drug delivery circuit. The CPAP systems currently in use at the study sites are single limb CPAP systems. Infants will be left on the F&P™ Bubble CPAP system for 30 min following the end of the first AeroFact^TM^ treatment, after which it will be determined if they meet re-dosing criteria to receive a second AeroFact^TM^ treatment. If the infant meets re-dosing criteria, they will remain on a F&P™ Bubble CPAP system for the duration of the second AeroFact^TM^ treatment. A new drug delivery circuit is required if the infant receives a second dose of aerosolized SF-RI 1, although the respiration sensor may remain in place. If an infant does not meet redosing criteria 30 min following completion of the first dose or following completion of the second dose of SF-RI 1, the drug delivery system and circuit and the respiration sensor will be removed from the infant’s bedside and the infant will be transitioned back to a standard flow driver CPAP device that is commonly used at the study site.

The CPAP strategy is the same during, between and after aerosolization. CPAP at 5–7 cmH_2_O is delivered to maintain peripheral oxygen saturation of 90–95%. CPAP and FiO_2_ will be weaned per study site unit protocol.

#### Control group

**Fig. 2:**
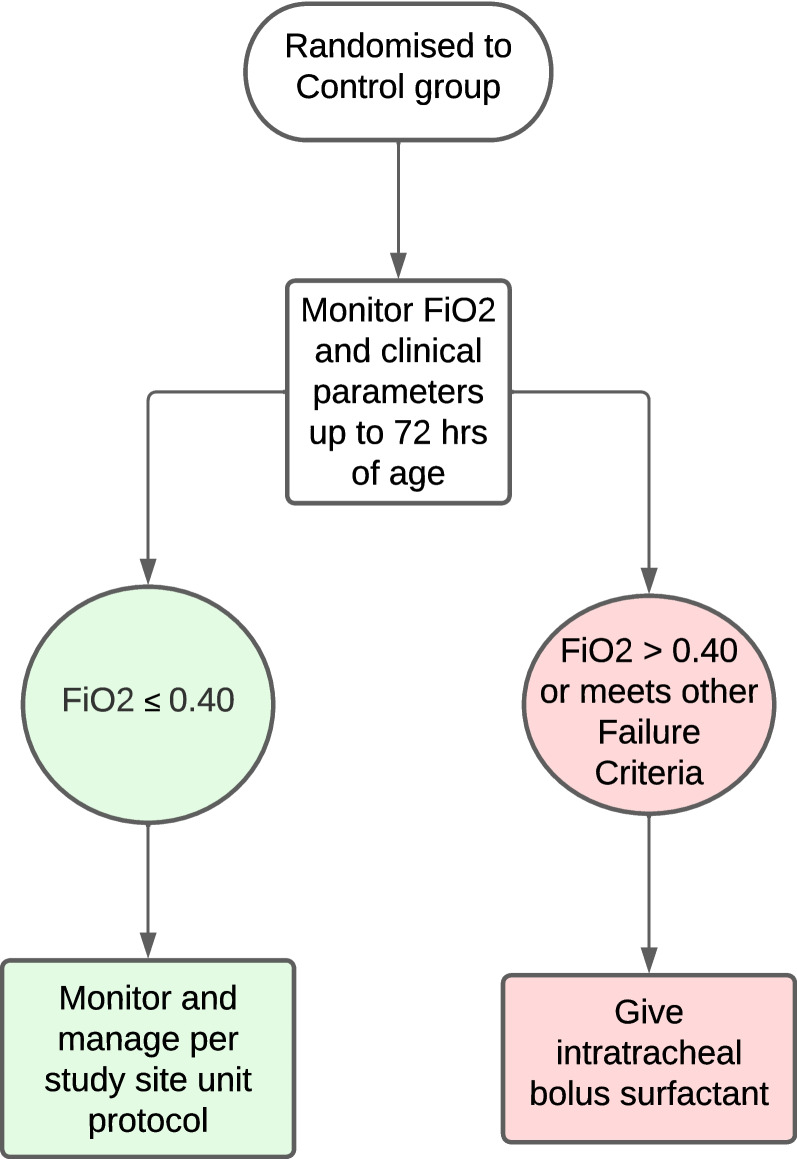
Control group flow chart

See Fig. 2 for control group flow chart. Infants randomised to the control group will continue to receive nCPAP alone with the standard flow driver CPAP device that is commonly used at the study site. There is no placebo or sham aerosolization in the control group as this is impractical in a resource-constrained setting and would have additional ethical implications. They will be eligible to receive intratracheal bolus surfactant if they meet failure criteria (see below).

#### Failure criteria

Infants in both the intervention and control groups will be eligible to receive intratracheal bolus surfactant instillation if they meet any of the following failure criteria at any time from randomisation to 72 h of age:

- A persistent FiO_2_ > 0.40 on nCPAP at 5–7cmH_2_O to maintain peripheral oxygen saturation of 90–95%. FiO_2_ requirement needs to be sustained for at least 15 min, and/or
- Severe apnoeas defined as two or more apnoeas per hour requiring bag-mask ventilation, or at the treating clinician’s discretion, and/or
- Severe increased work of breathing (at the treating clinician’s discretion) not responding to nCPAP optimization, repositioning of infant or suctioning of secretions.

At each site, an FiO_2_ > 0.40 is consistent with the unit protocol to administer intratracheal bolus surfactant and aligns with the current South African standard of care. Beractant 100 mg/kg (Survanta) or Poractant Alfa 100 mg/kg (Curosurf) may be administered by LISA, INSURE or intubation for surfactant delivery followed by mechanical ventilation. The method of administration and the choice of surfactant used is at the discretion of the treating clinician.

#### Concomitant therapy

At all sites, all infants < 32 weeks’ gestation will receive a loading dose of caffeine on admission to the neonatal unit, and a maintenance dose as prophylaxis for apnoea of prematurity. Caffeine will be discontinued if infants have reached 34 weeks’ gestation and have had no apnoeas for one week. Caffeine continuation beyond 34 weeks’ gestation is at the treating clinician’s discretion and reasons for continuation must be appropriately documented. Only caffeine use will be standardised across all three sites. All other concomitant therapies used in neonatal medicine are allowed and their use is indicated per study site protocol. These concomitant therapies do not have to be standardised across sites but must be the same between intervention and control groups at each site.

#### Outcomes

The primary outcome is the proportion of infants requiring intratracheal bolus surfactant administration in the first 72 h of age in each study group.

Secondary outcomes include:

- Proportion of infants meeting failure criteria in the first 72 h of age in each group
- Proportion of infants receiving multiple doses of intratracheal bolus surfactant in each group
- Number of hours on mechanical ventilation during hospital stay between groups
- Number of days on CPAP and/or HFNC, and supplementary oxygen during hospital stay between groups
- Incidence of adverse events and select comorbidities of prematurity between groups (see Appendix for definitions)
- Incidence of BPD in survivors at 36 weeks’ postmenstrual age between groups (see Appendix for definitions)
- Incidence of BPD or death at 36 weeks’ postmenstrual age between groups

#### Participant timeline

**Fig. 3:**
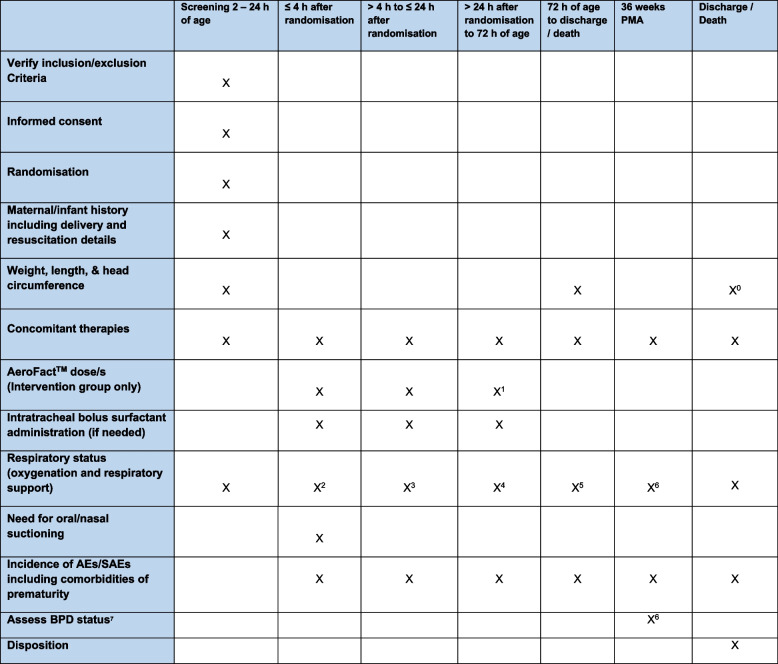
Schedule of assessments. ^0^ Weight and head circumference only (excluding length). ^1^ AeroFact^TM^ dose/s only up to 32 h of age. ^2^ Respiratory Status assessed every 30 min and other clinically relevant timepoints. ^3^ Respiratory Status assessed every hour and other clinically relevant timepoints. ^4^ Respiratory Status assessed every 3 h and other clinically relevant timepoint. ^5^ Respiratory Status assessed daily. ^6^ Respiratory Status assessed by telephone if transferred to another hospital prior to 36 weeks postmenstrual age. ^7^ See Appendix for definitions

See Fig. 3 for schedule of assessments. If an infant receives intratracheal bolus surfactant, the following information will be documented:

- Date, time, and age of administration
- Surfactant type, dosing details and method by which it was given (LISA, INSURE, etc.), and
- Respiratory status, including FiO_2,_ oxygen saturation and type of respiratory support, at the time of administration

If an infant meets failure criteria the age at which the infant met failure criteria will be documented.

Serial measurements of respiratory status will be collected at various timepoints from birth to discharge home or death and will include:

- Need for oral/nasal suctioning
- Respiratory status including type and level of support, FiO_2_ requirement and peripheral oxygen saturation

Additional data on the clinical course in hospital will be collected and include the following:

- The incidence of adverse events and selected comorbidities of prematurity (see Appendix for definitions)
- BPD status assessed at 36 weeks’ postmenstrual age (see Appendix for definition)
- Discharge disposition

Infants who are transferred to another hospital will be followed up by study staff to ascertain their BPD status (if transferred prior to 36 weeks’ postmenstrual age) and discharge disposition.

#### Sample size

The proportion of infants who will require intratracheal bolus surfactant administration in the control group is estimated to be 45%. Assuming a 40% relative risk reduction to reduce the proportion of infants requiring intratracheal bolus surfactant to 27%, the study will aim to enrol approximately 220 infants (110 infants in each arm). To accommodate for a 5% dropout rate, we will aim to enrol approximately 232 infants (116 infants in each arm) for the study to have a power of 80% to detect a significant difference with a type 1 error of 0.05.

#### Recruitment

**Fig. 4:**
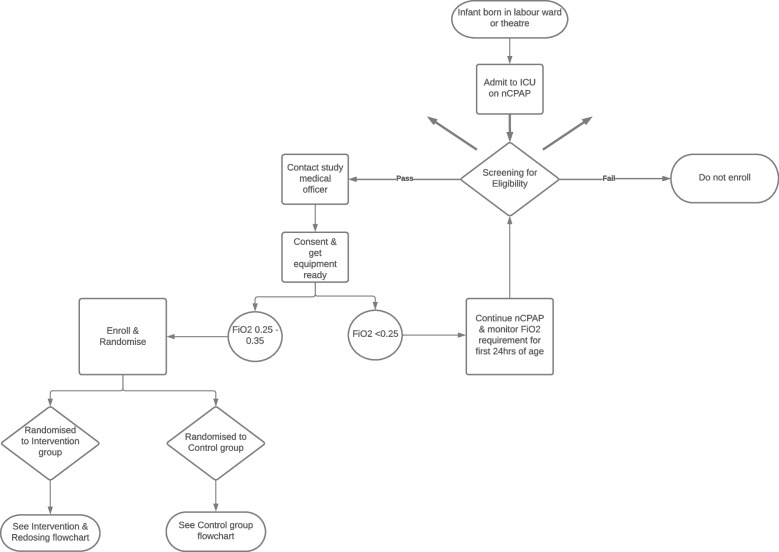
Study enrolment flow chart

See Fig. 4 for study enrolment flow chart. All potential study participants will be recruited by dedicated study staff in the NICU and/or high care area at each site. Non-study clinical staff may inform the study staff of potential study participants. Infants will be screened from 2 to 24 h of age to determine if they meet eligibility criteria.

The mother of an eligible infant will be approached by a study staff member to describe the nature of the study and the proposed benefits, and the potential risks associated with study participation. An opportunity to ask questions will be provided. Once all questions and concerns have been addressed by the study staff member, written informed consent will be obtained. See supplementary material for model informed consent form. A copy of the signed informed consent will be provided to the mother of the infant. Mothers who consent for their infants to take part in the study will be reimbursed for their time, inconvenience, and expenses as per the South African Health Products Regulatory Authority (SAHPRA) suggested compensation model.

### Assignment of interventions

#### Allocation

Randomisation will occur strictly at 2–24 h of age. A web-based system will randomise the eligible infant in a 1:1 manner to receive aerosolized surfactant while on nCPAP (intervention group) or continue on nCPAP alone (control group). The computer-generated, balanced-block randomisation sequence will be stratified by site and by weight band (900–1199 g and 1200–1999 g). If twins are enrolled, each infant will be randomised independently.

#### Blinding

The study staff, NICU staff and infant’s mother will not be blinded to the study group allocation. However, several aspects of the study protocol are designed to reduce bias, including:

- Objective failure criteria to be eligible for intratracheal bolus surfactant, based on FiO_2_ requirements. These are collected as a secondary endpoint.
- Consistent policies for use of caffeine between groups at all sites
- Consistent peripheral oxygen saturation targets which drive duration of supplemental oxygen use between groups at all sites
- Consistent criteria for diagnosis of BPD at all sites

### Data collection, management, and analysis

#### Data collection methods

Trained study staff will enter the information from the infant’s hospital record and source documentation as required by the protocol onto the electronic case report form (eCRF) in accordance with the eCRF completion guidelines. See supplementary material for complete CRF. An infant who is withdrawn from the study for safety or administrative reasons will immediately stop any aerosolization (if in the intervention group) and will have no further data collected for study purposes. However, the infant will remain in the study for data analyses and outcomes measurements, unless the mother specifically requests that their infant’s data not be included.

#### Data management

Quality control and data validation procedures will be applied to ensure the validity and accuracy of the clinical database.

#### Statistical methods

The primary analysis will be the Intention-to-Treat (ITT) population, defined as all randomised infants, regardless of their adherence with the entry criteria, treatment they received or subsequent withdrawal from treatment, or deviation from the protocol. The Per-Protocol (PP) population is defined as all randomised infants who were not associated with any major protocol violations. The Safety Population will include all subjects who are randomised and started their allocated treatment.

All study data collected onto the eCRF will be exported for statistical analyses. Continuous variables will be summarized by treatment group and overall using descriptive statistics (n, mean, standard deviation, median, interquartile range, minimum and maximum). Frequencies and percentages will be presented by study group and overall, for categorical variables. All inferential statistical analysis will be based on a two-sided test with a type I error of 0.05. The efficacy analyses of the primary and secondary efficacy endpoints will be conducted on the ITT population. The PP population analyses of primary and secondary endpoints will be considered supportive.

#### Data monitoring

Each site will receive rigorous in-person monitoring of protocol compliance and study data by a clinical research associate (CRA) from OnQ Research, a clinical research organisation. CRAs will periodically visit each investigational site to review the eCRFs for completeness and accuracy against the source documents. CRAs will highlight any discrepancies found in the documentation of study processes and ensure that appropriate site personnel address the discrepancies. CRAs will also perform investigational product (IP) accountability during the study and prior to study close-out.

#### Harms

Safety will be assessed by comparison of the rate of adverse events (AE) between the intervention and control groups. AEs for this study include select co-morbidities of prematurity (see supplementary material) or any untoward medical occurrence deemed significant by the site principal investigator (PI). An AE does not necessarily have to have a causal relationship with the IP. The PI will be asked to determine the causal relationship of each AE to the investigational drug/device. All AEs will be coded using the Medical Dictionary for Regulatory Affairs and SAEs will be summarized by treatment group, severity, and relationship to study treatment.

An independent Data Safety and Monitoring Board (DSMB) will consist of at least three neonatologists and one statistician who are not investigators in the study nor otherwise associated with the study. The DSMB will periodically review and evaluate the accumulated study data for participant safety, study conduct and progress, and make recommendations concerning the continuation, modification, or termination of the trial for safety purposes. The DSMB will establish its own charter, procedures, and criteria for recommendations regarding safety.

#### Audit

The database will be reviewed and checked for omissions, apparent errors, and values requiring further clarification using computerized and manual procedures. Data queries requiring clarification will be documented and returned to the study site for resolution. Only authorized personnel will make corrections to the clinical database, and all corrections will be documented in an audit trail.

### Supplementary Information

**Additional file 1.** SPIRIT 2013 Checklist: Recommended items to address in a clinical trial protocol and related documents.

**Additional file 2: Appendix A.** Definitions. **Appendix B.** Bacterial pathogens.

**Additional file 3. **Informed Consent Form.

**Additional file 4. **Case Report Form.
