# Supplementary material for: A multicentre neonatal interventional randomised controlled trial of nebulized surfactant for preterm infants with respiratory distress: Neo-INSPIRe trial protocol
Source: BMC Pediatr. 2023 Sep 19;23:472. doi: 10.1186/s12887-023-04296-4 (PMC10507916; doi:10.1186/s12887-023-04296-4)
Supplement: Supplementary file 2 — Additional file 2: Appendix A. Definitions. Appendix B. Bacterial pathogens. [file 12887_2023_4296_MOESM2_ESM.pdf]

APPENDIX A - DEFINITIONS

Note: Definitions adapted from Vermont Oxford Network and The International Neonatal Consortium, Neonatal Adverse Event Severity Scale (INC NAESS)

(Available at: https://vtoxford.zendesk.com/hc/en-us/articles/4405064008467-2022-Manual-of-Operations-Part-2-Release-26-2-PDF- and https://pubmed.ncbi.nlm.nih.gov/31537552/)

The following are documented as adverse events in the **first 72 hours of age ONLY:**

BRADYCARDIA

- Heart rate < 80 beats per minute sustained for at least 30 minutes.

TACHYCARDIA

- Heart rate > 200 beats per minute sustained for at least 30 minutes.
- Tachycardia NOT related to atropine use, hyperthermia or another confirmed adverse event such as infection.

The following are documented as adverse events at **ANY time during admission (**i.e., from randomisation until discharge/death)

BACTERIAL SEPSIS AND/OR MENINGITIS

- One or more of the following bacterial pathogens was recovered from a blood and/or cerebrospinal fluid culture obtained.
- Up to three bacterial pathogens may be recorded.
- See list of pathogens in Appendix B

BRONCHOPULMONARY DYSPLASIA (BPD)

A preterm infant < 33 weeks gestation will be deemed to have BPD if they require respiratory support at 36 weeks PMA (adapted from VON criteria and Bronchopulmonary Dysplasia: Executive Summary of a Workshop (Higgens et al (16)). BPD will be graded as follows:

**Grades**	**Invasive MV**	**nCPAP or HFNC > 2L/min**	**NC ≤ 2L/min**
**I**		21 %	22 – 29 %
**II**	21 %	22 – 29 %	≥ 30 %
**III**	> 21 %	≥ 30 %	

Abbreviations: MV = mechanical ventilation; nCPAP = nasal continuous positive airway pressure; HFNC = high flow nasal cannula; NC = nasal cannula; min = minutes

COAGULATION DISORDER

- Abnormal blood clotting or bleeding that requires intervention with blood products.

PERIVENTRICULAR – INTRAVENTRICULAR HAEMORRHAGE (PIH), WORST GRADE

- If a cranial ultrasound was performed on or before Day 28, the worst grade of PIH will be documented using the criteria below.

    - Grade 1: Subependymal germinal matrix haemorrhage only
    - Grade 2: Intraventricular blood, no ventricular dilation
    - Grade 3: Intraventricular blood, ventricular dilation
    - Grade 4: Intraparenchymal haemorrhage

FOCAL INTESTINAL PERFORATION

- Surgically confirmed based on visual inspection of the bowel at the time of surgery or post-mortem examination that demonstrates a single focal perforation with the remainder of the bowel appearing normal, or clinically confirmed if they do not satisfy the definition for NEC.

FUNGAL INFECTION

- Document if a fungus was recovered from a blood culture obtained from either a central line or peripheral blood sample.

RETINOPATHY OF PREMATURITY (ROP)

If a retinal examination was performed, document the worst stage on any exam in the eye with the most advanced stage.

  - Stage 1: Presence of demarcation line (+/- abnormal vascularization)
  - Stage 2: Presence of intraretinal ridge
  - Stage 3: Presence of a ridge with extraretinal fibrovascular proliferation
  - Stage 4: Partial retinal detachment
  - Stage 5: Total retinal detachment

NECROTIZING ENTEROCOLITIS (NEC)

Must be diagnosed at surgery, at post-mortem examination, or with clinical and diagnostic imaging using the following criteria.

- At least one of the following clinical signs present:
  - Bilious gastric aspirate or emesis
  - Abdominal distension or discoloration
  - Occult or gross blood in stool (no fissure),

AND

- At least one of the following diagnostic imaging findings present:
    - Pneumatosis intestinalis
    - Hepato-biliary gas
    - Pneumoperitoneum

PATENT DUCTUS ARTERIOSUS (PDA)

- At least one of the following findings is present
    - Left to Right or bidirectional ductal shunt on Doppler echo
    - Systolic or continuous murmur

AND

- At least two of the following findings are present:
    - Hyperdynamic precordium
    - Bounding pulses
    - Wide pulse pressure
    - Pulmonary vascular congestion, cardiomegaly, or both

PERIVENTRICULAR – INTRAVENTRICULAR HAEMORRHAGE (PIH), WORST GRADE

- If a cranial ultrasound was performed on or before Day 28, the worst grade of PIH will be documented using the criteria below.

    - Grade 1: Subependymal germinal matrix haemorrhage only
    - Grade 2: Intraventricular blood, no ventricular dilation
    - Grade 3: Intraventricular blood, ventricular dilation
    - Grade 4: Intraparenchymal haemorrhage

PNEUMOTHORAX

- The presence of extra-pleural air diagnosed by chest radiograph or needle aspiration (thoracentesis)

PULMONARY HAEMORRHAGE

- Frank blood with respiratory compromise requiring escalation of respiratory support.

SEIZURES

- Record seizures as adverse events only if:
  - Status epilepticus: > 30 minutes duration within any hour, OR
  - Recurrent seizures: each > 30 seconds per hour requiring > 1 anti-seizure drug within a 3-day period.
- Seizures should preferably be EEG confirmed.
- Can be electro-clinical or electrographic only.

SEVERE APNOEA

- Two or more apnoeas per hour requiring bag-mask ventilation.

SEVERE HYPOTENSION

- Requiring inotropes to maintain blood pressure.

APPENDIX B

BACTERIAL PATHOGENS

- Achromobacter species [including A. xylosoxidans (also known as Alcaligenes xylosoxidans) and others]
- Acinetobacter species including multidrug-resistant Acinetobacter
- Aeromonas species
- Alcaligenes species [A. xylosoxidans and others]
- Bacteroides species
- Burkholderia species [B. capecia and others]
- Campylobacter species [C. fetus, C. jejuni and others] including drug-resistant Campylobacter
- Chryseobacterium species
- Citrobacter species [C. diversus, C. freundii, C. koseri and others]
- Clostridium species
- Enterobacter species [E. aerogenes, E. cloacae, and others] including Carbapenem-resistant Enterobacter
- Enterococcus species [E. faecalis (also known as Streptococcus faecalis), E. faecium, and others] including Vancomycin-resistant Enterococcus
- Escherichia coli including Carbapenem-resistant Escherichia coli
- Flavobacterium species
- Haemophilus species [H. influenzae and others]
- Klebsiella species [K. oxytoca, K. pneumoniae and others] including Carbapenem-resistant Klebsiella and Cephalosporin-resistant Klebsiella
- Listeria monocytogenes
- Moraxella species [M. catarrhalis (also known as Branhamella catarrhalis) and others]
- Morganella morganii
- Neisseria species [N. meningitidis, N. gonorrhoeae and others] including drug- resistant N. gonorrhoeae
- Pantoea
- Pasteurella species
- Prevotella species

- Proteus species [P. mirabilis, P. vulgaris and others]
- Providencia species [P. rettgeri and others]
- Pseudomonas species [P. aeruginosa and others] including multidrug-resistant Pseudomonas aeruginosa
- Ralstonia species
- Salmonella species including drug-resistant Salmonella serotype Typhi
- Serratia species [S. liquefaciens, S. marcescens and others]
- Staphylococcus coagulase positive [aureus] including Methicillin-resistant Staphylococcus aureus and Vancomycin-resistant Staphylococcus aureus
- Stenotrophomonas maltophilia
- Group B Streptococcus or GBS [also known as Streptococcus agalactiae]
- Streptococcus anginosus [formerly Streptococcus milleri]
- Streptococcus pneumoniae
- Streptococcus pyogenes [Group A Streptococcus]
