# Supplementary material for: A multicentre neonatal interventional randomised controlled trial of nebulized surfactant for preterm infants with respiratory distress: Neo-INSPIRe trial protocol
Source: BMC Pediatr. 2023 Sep 19;23:472. doi: 10.1186/s12887-023-04296-4 (PMC10507916; doi:10.1186/s12887-023-04296-4)
Supplement: Supplementary file 3 — Additional file 3. Informed Consent Form. [file 12887_2023_4296_MOESM3_ESM.pdf]

INFORMATION AND INFORMED CONSENT FORM FOR PARENTS OF INFANT PARTICIPANTS

**Protocol no.**<br><br>**Study Name:**	APC-AF-CLN-004<br><br>NEO-INSPIRe
**Protocol title**	A South African, multicenter, phase 2, non-blinded, randomized controlled trial to determine if aerosolized surfactant plus continuous positive airway pressure (CPAP) compared to CPAP alone can improve the course of infants with respiratory distress syndrome (RDS) by decreasing their need for intratracheal bolus surfactant in the first 72 hours of age.
**Protocol Short Title**	Neonatal Interventional Nebulized Surfactant for Preterm Infants with RDS
**Sponsor**	Aerogen Pharma Limited
**Principal Investigator**	**Prof Lloyd Tooke**<br>Groote Schuur Hospital Neonatal Intensive Care Unit, Groote Schuur Hospital, G Floor, Anzio Road, Observatory, Cape Town, 7925, South Africa
**Day and After-Hours Telephone Number**	**DAY:** +27 21 404 6022<br><br>**AFTER HOURS:**

Subject ID number:

Mom's sticker:

INTRODUCTION

Good day, my name is ………………………………………………………………….. (Insert name of study doctor), I am a ………………………………………………………… (Insert designation) at **Groote Schuur Hospital Neonatal Unit**.

I would like to invite you to consider your baby participating in a research study, entitled NEO-INSPIRe. Before you agree to participate, it is important that you read and understand the following explanation of the purpose of the study, the study procedures, benefits, risks, discomforts, and precautions as well as the alternative procedures that are available for your baby, and your right to withdraw from the study at any time.

- This Information and Informed Consent Form is to help you to decide if you would like your baby to participate. You need to understand what is involved before you agree to take part in this study.
- If you have any questions, do not hesitate to ask me.
- You should not agree to take part unless you are satisfied with all the procedures involved.
- If you decide that your baby may take part in this study, you will be asked to sign this document to confirm that you understand the study. You will be given a copy to keep.

WHAT IS THE RESEARCH STUDY ABOUT?

Babies that are born early or before their due date (usually before 34 weeks) may have difficulty breathing when they are born. Their lungs have not had enough time to develop properly, and they cannot yet make surfactant. Surfactant is an important substance that is made by the lungs (usually after 34 weeks) and it helps keep the small air-sacs (alveoli) of the lung open. When the air-sacs are open, breathing is easy. When they are closed or collapsed, breathing can be very hard, a bit like blowing up a balloon. This disease is called Respiratory Distress Syndrome (RDS) and it usually develops in the first 24 hours after the premature baby is born.

There are a few known ways to help small babies with RDS breathe when they are born. The first is using nasal Continuous Positive Airway Pressure (nCPAP) which blows air into the small airways or air-sacs to help keep them open. Most premature babies who have difficulty breathing when they are born will be put onto nCPAP almost immediately. If small babies continue to struggle to breath even on nCPAP, we can give them liquid surfactant which is taken from a cow or a pig's lungs. Giving surfactant to a small baby is done by inserting a very small feeding tube into the entrance of their airways and slowly injecting the liquid surfactant into the tube so that it can reach the lungs. If a baby continues to struggle to breath a breathing tube can be inserted into the airways and a machine (ventilator) breathes for the baby. Liquid surfactant can also be given down the breathing tube, if necessary.

WHAT IS THE PURPOSE OF THE STUDY?

You are being invited to the study because your baby was born early and is currently admitted in the Neonatal ICU (NICU) and has been diagnosed with RDS. Your baby meets all the criteria required to be enrolled in this study. The inclusion criteria are:

- Baby born at our hospital
- Baby weighs 900 – 1999 grams
- Baby is between 27 – 34 weeks gestation
- Baby is between 2 – 24 hours old
- Baby is receiving oxygen and is on nCPAP for breathing difficulties

The study is an experimental research study which aims to see if there is an easier way to give surfactant to premature babies with breathing difficulties. 232 babies will be randomly assigned, so that half of them receive nebulised surfactant while on nCPAP and the other half continue on nCPAP without receiving nebulised surfactant. We will compare the two groups to see if nebulised surfactant decreases the need for surfactant given by a feeding tube or breathing tube.

WHAT IS NEBULISED SURFACTANT?

A nebuliser machine converts a liquid medication into a fine mist. This is similar to the nebuliser that people with asthma use when they need to inhale medication for a tight chest. Rather than using a feeding or breathing tube to give the liquid surfactant, the nebuliser changes the liquid surfactant into fine mist (aerosol) which can be inhaled by the baby directly into their lungs.

WHY IS THIS STUDY IMPORTANT?

Although the doctors looking after your baby perform these techniques almost every day and are very skilled at doing it, placing a tube into a small baby's airways can be a difficult procedure that requires a lot of practice. Sometimes complications can occur during the procedure; the liquid surfactant can go into the stomach by accident rather than the lungs, or the baby's oxygen levels, and heart rate may drop very low. If this study shows that giving nebulised surfactant to preterm babies with breathing difficulties does prevent them from needing liquid surfactant given through a tube, more babies that live in remote and rural areas may be able to receive this treatment. Nebulising surfactant is an easy process and the nurse or doctor giving the nebulised surfactant does not need to be skilled or trained in giving surfactant through a tube, which can be a difficult procedure in small babies.

WHAT KIND OF SURFACTANT IS USED IN THIS STUDY?

This study uses "**SF-RI 1"** which is a natural surfactant taken from a cow's lungs and is made in Germany. It is given as liquid surfactant by a feeding or breathing tube to treat babies with Respiratory Distress Syndrome, in 30 countries of the world. It is not, however, approved for use in South Africa yet. It is also not yet approved anywhere in the world to give SF-RI 1 via nebulising, and this is what we are aiming to test.

WHAT KIND OF NEBULISER IS USED IN THIS STUDY?

The sponsor of this study, Aerogen Pharma Limited, has developed an experimental drug and delivery device combination called **"AeroFact"**. It nebulises SF-RI 1 surfactant and delivers the drug in a fine mist to the nCPAP machine that the baby is on.

WHAT WILL HAPPEN IN THE STUDY?

If you provide consent for your baby to take part in this study, we will enrol your baby in the study. Your baby will be given a unique study number and then will be randomly allocated to one of two groups: the treatment group or the control group. There is a 50/50 chance of being allocated to either one of the groups. This is a non-blinded study which means that you, the study staff, and the hospital staff will know which group your baby has been allocated to.

IF YOUR BABY IS ALLOCATED TO THE TREATMENT GROUP:

Your baby will already be on nCPAP when they are enrolled in the study. If they meet the Intervention criteria, they will be connected to the "Aerofact" machine to receive one dose of nebulised surfactant via the CPAP machine. They will be monitored carefully to ensure their safety and to determine if they need liquid surfactant via a feeding or breathing tube.

When they have completed one dose of nebulised surfactant, the study staff member will assess your baby and decide if they can receive one more dose of nebulised surfactant. Your baby can only receive two doses of nebulised surfactant from the time they are enrolled until they are 32 hours old. The nCPAP will be stopped only when the doctor treating your baby decides it is safe to do so.

IF YOUR BABY IS ALLOCATED TO THE CONTROL GROUP:

Your baby will already be on nCPAP when they are enrolled in the study. They will not receive nebulised surfactant and they will be managed in the same way as they would usually be managed in the NICU/High Care Area. This is called the standard of care. We use the results of the babies in the control group to compare them to the results of the babies of the AeroFact group. This is to test if nebulised surfactant is safe and effective. They will be monitored carefully to determine if they need liquid surfactant via a thin catheter or breathing tube. The nCPAP will be stopped only when the doctor treating your baby decides it is safe to do so.

All other medication and therapies used to treat preterm babies will be the same in both groups and your baby will be treated as they usually are in the NICU/High Care Area.

NUMBER OF PARTICIPANTS AND LENGTH OF STUDY:

The study will be performed in South Africa only and at three sites and approximately 232 babies will participate in this study. Babies are enrolled in the study from day 1 of life until they are discharged home. They can only receive the study treatment in the first 32 hours of life. After 32 hours of life, we will continue to collect important data from your baby. All preterm babies are at risk of developing complications of prematurity, and we need to monitor and document these.

PROCEDURES

Once your baby is enrolled, we will collect data from the hospital folder which includes your antenatal history, information about the delivery, baby's weight, length and head circumference and any medication your baby may be on. We will also look in detail at how your baby is doing during their hospital stay - what kind of support they need for their breathing difficulties, how much oxygen they require and how they are responding to the treatment. We will collect this data from the time they are enrolled until they go home. It will not interfere in any way with their normal care.

WHAT ARE THE POTENTIAL BENEFITS TO MY BABY IN TAKING PART IN THIS STUDY?

There may not be any benefit from participating in this study. However, if nebulised surfactant is effective for treating babies with RDS, then your baby may not need to have liquid surfactant administered by a thin catheter or breathing tube. This reduces their risks of the complications that are associated with this procedure.

WHAT ARE THE POTENTIAL RISKS TO MY BABY IN TAKING PART IN THIS STUDY?

Several other studies have shown that giving nebulised surfactant is safe and well tolerated, but no procedure is without any risk. The nebulised surfactant may not work, and your baby may still require a thin catheter or breathing tube to receive liquid surfactant. The inhaled fine mist may cause a blocked or runny nose which can interfere with breathing. The study staff will monitor your baby closely and will suction the nose if necessary. The tape used to secure a breathing sensor may cause some irritation. The study staff will monitor the area closely and use a protective wipe to remove the tape to limit pain and damage to the skin.

UNFORESEEN RISKS

Unforeseen risks are risks to your baby and side effects from the study treatment that occur that we do not expect or have any knowledge of. We will aim to reduce the risk and ensure the safety of your baby at all times by monitoring them closely.

COMPENSATION

You will not be paid to take part in this study, but you will be compensated for your time and inconvenience according to the South Africa Health Products Regulatory Authority (SAHPRA)

guidelines. You will be given R150.00 South African Rand in electronic cash vouchers that will be sent to your cell phone.

RIGHTS AS A PARTICIPANT IN THIS STUDY

If you decide not to take part in this study you will still receive the best current care, from your usual doctor. Your baby's participation in this study is entirely voluntary and you can decline to participate, or stop at any time, without stating any reason. The withdrawal will not affect access to other medical care. If you do not wish to provide consent, your baby will not be enrolled in the study, and they will continue to receive standard quality of care by the doctors and nurses in the NICU/High Care Area.

WITHDRAWAL

You may withdraw consent at any time if you choose to. You do not need to provide a reason if you prefer not to. A study staff member will ask if you choose to withdraw consent from the study treatment only or from having any further data collected. Withdrawing consent from the study will not have any impact on the standard quality of care your baby receives by the doctors and nurses.

NEW FINDINGS

You have the right to be informed about any new information about nebulised surfactant that becomes available during the study. The sponsor of the study, Aerogen Pharma Limited, may choose to stop the study early. You can also choose to withdraw consent from the study based on any new information that becomes available during the study.

WILL I BE TOLD ABOUT THE RESULTS OF THE STUDY?

A description of this study will be available on http://www.sanctr.gov.za as required by US and South African law. These websites will not include information that can identify you. The websites will include a summary of the final study results. You can access these websites at any time. If you want the results to be made available to you, please talk to the study staff.

We will publish the results of this in medical journals so that other doctors can learn about it. These reports will not include any of your personal information (for example your name or where you live). Your baby's data may be used if it is needed only for the study and may be kept for longer, where required, by law. Study records will be kept for 10 years from the end of the study.

FINANCIAL ARRANGEMENTS

You are not expected to pay any money for your baby to participate in this study. The sponsor, Aerogen Pharma, will pay for all the study procedures.

INSURANCE

Aerogen Pharma Limited has obtained insurance for your baby in the event of study related injury or illness. A study-related injury or illness is one that occurs as a direct result of the administration of the study medicine or of study-specific procedures. Aerogen Pharma Limited will provide compensation for reasonable medical expenses incurred because of study-related injury or illness.

The insurance does not cover, and Aerogen Pharma Limited will not pay for:

- Medical treatment of the other injuries or illnesses
- Injury caused if protocol is not followed.

Name of the insurer – Chubb Insurance South Africa Limited

ETHICAL APPROVAL

This clinical study protocol has been approved by the Human Research Ethics Committee (HREC) at the University of Cape Town.

WHO CAN I SPEAK TO IF I HAVE ANY QUESTIONS OR COMPLAINTS AFTER MY BABY IS ENROLLED?

You can direct any questions or complaints you have during the study to your baby's study doctor, the Principal Investigator at Groote Schuur Hospital or the UCT ethics committee.

Other doctors from this department who are working on this study are:

Dr Elizabeth Lategan and Dr Michael Harrison.

The 24-hour telephone number by which you can reach me or another authorised person:

The UCT Faculty of Health Sciences Human Research Ethics Committee can be contacted on 021 406 6338 in case you have any ethical concerns or questions about your baby's rights or welfare as a participant in this research study.

After you have consulted your baby's study doctor or the ethics committee and if they have not provided you with answers to your satisfaction, you should write to SAHPRA at:

The Chief Executive Officer
Dr Boitumelo Semete-Makokotlela
South African Health Products Regulatory Authority
Department of Health
Private Bag X828
PRETORIA
0001
Tel: (012) 501 0410
Email: Boitumelo.Semete@sahpra.org.za

CONFIDENTIALITY

All information obtained during this study, including hospital records, personal data and research data will be kept strictly confidential. Data that may be reported in scientific journals will not include any information that identifies your baby as a participant in this study. Your baby will have a study number allocated and the name of you and your baby will not be documented anywhere in the database. The data is all collected electronically (on a laptop or tablet), and these devices are protected by a security password known only to the study staff. The information will be stored in the cloud and is only accessible to the Aerogen Pharma Limited, specific study staff members and study monitors who ensure that the study is being performed correctly. This information will be reviewed by authorised representatives of the Aerogen Pharma Limited.

The information might also be inspected by the National Health Research Ethics Council (NHREC), University of Cape Town Human Research Ethics Committee (HREC), SAHPRA and/or other regulatory bodies. Therefore, you hereby authorise me to release your baby's medical records to the Aerogen Pharma Limited, its employees or agents, domestic and foreign regulatory health authorities, and UCT HREC. These records will be used by them only in connection with carrying out their work relating to this clinical study.

Your baby's anonymous data may be analysed and, used for future research that may not be related to the study only if HREC approval is granted for this purpose. Your baby's anonymous data may also be analysed and used for submission to governmental authorities, for legal or regulatory purposes, or to seek approval of a medicine or treatment for marketing from governmental authorities.

PARTICIPANT QUESTIONS

DID THE PARTICIPANT'S PARENT RAISE ANY QUESTIONS: YES / NO

If YES – What were they:

____

____

____

____

____

INFORMED CONSENT:

- ……………………………………………………………………………. *(Insert name of study doctor)* has provided me with a copy of the Information and Informed Consent Form regarding clinical study, APC-AF-CLN-004: A South African multicenter, phase 2, non-blinded, randomized controlled trial to determine if aerosolized surfactant plus continuous positive airway pressure (CPAP) compared to CPAP alone can improve the course of infants with respiratory distress syndrome (RDS) by decreasing their need for intratracheal bolus surfactant in the first 72 hours of age and has fully explained to me the nature, risks, benefits and purpose of the study.
- The study doctor has given me the opportunity to ask any questions concerning both the medicine and the study.
- It has been explained to me that I will be free to withdraw my baby from the study at any time, without any disadvantage to future care.
- I have understood everything that has been explained to me and I consent for my baby to participate in this clinical study.

MOTHER:

**Printed Name of the mother:**	**Signature / Mark or Thumbprint**	**Date and Time**

STUDY DOCTOR:

I hereby confirm that the above participant's representative has been fully informed about the nature, conduct and risks of the above study.

**Printed Name of the Study Doctor:**	**Signature**	**Date and Time**

TRANSLATOR (if applicable)

**Printed Name of the Translator**	**Signature**	**Date and Time**

WITNESS (If applicable): Only required if no translator present

**Printed Name of the Witness**	**Signature**	**Date and Time**
