# Supplementary material for: A multicentre neonatal interventional randomised controlled trial of nebulized surfactant for preterm infants with respiratory distress: Neo-INSPIRe trial protocol
Source: BMC Pediatr. 2023 Sep 19;23:472. doi: 10.1186/s12887-023-04296-4 (PMC10507916; doi:10.1186/s12887-023-04296-4)
Supplement: Supplementary file 4 — Additional file 4. Case Report Form. [file 12887_2023_4296_MOESM4_ESM.pdf]

APC-AF-CLN-004 Source Documents

Table of Contents

Screening 2 – 24 hr. of Age:
Form 1 – Screening and Eligibility - Informed Consent and Inclusion/Exclusion Criteria
Form 2 – Randomization and Infant Demographics
Form 3 - Infant Delivery Information
Form 4 - Maternal/Perinatal Data

Birth-Growth Log:
Form 5 – Birth Infant Measurements and Growth

Dosing (1, 2):
Form 6 - AeroFact Dosing

Serial Respiratory Status Through Discharge/Death:
Form 7a - Serial Respiratory Status ≤ 4 h after randomization
Form 7b - Serial Respiratory Status > 4 h to 24 h after randomization
Form 7c - Serial Respiratory Status > 24 h to 72 h after randomization
Form 7d - Serial Respiratory Status 72 after randomization to Discharge/Death

Treatment Failure:
Form 8 - Treatment Failure

Intubations and Extubations Log
Form 9 - Intubations and Extubations Log

Bolus Surfactant Instillation Log (1, 2, 3)
Form 10 - Bolus Surfactant Instillation Log

36 Weeks PMA:
Form 11 - 36 Week Respiratory and BPD Status

Adverse Events:
Form 12 - Adverse Events

Surgeries:
Form 13 - Surgeries

Transfusions :
Form 14 - Transfusions

Concomitant Medications :
Form 15 - Concomitant Medications

Discharge:
Form 16 - Discharge from NICU

Protocol Deviations :
Form 17 - Protocol Deviations

Device Performance :
Form 18 - Device Performance

Participant Number: _______________ Site Number: ______

Form 1 *(page 1)*

Patient Eligibility 2 – 24 hrs. of Age

1.0. Inclusion/Exclusion

Infants must have a **YES** box checked for each of the following criteria for study inclusion:

#	Inclusion Criteria	YES	NO
1	Inborn		
2	Birth Weight 900 – 199 g		
3	27-34 weeks gestational age		
4	2 – 24 hours of age at the time of randomization		
5	Persistent fractional inspired oxygen (FiO2) of 025 – 0.35 on nCPAP at 5-7 cmH2O to maintain peripheral oxygen saturation of 90% -95%. FiO2 requirement needs to be sustained for at least 15 minutes.		

If **NO** is answered to any of the above, the infant is a screen failure and will not continue in the study.

Infants must have a **NO** box checked for each of the following criteria for study inclusion:

#	Exclusion Criteria	YES	NO
1	Administration of inotropes and/or intubation prior to enrollment (in the delivery room or the NICU)		
2	5-minute APGAR score $\leq$ 5		
3	Prior instillation of intratracheal surfactant		
4	Pneumothorax that requires needle thoracentesis or insertion of intracostal chest drain		
5	Life threatening congenital anomaly		
6	Known or suspected chromosomal abnormality		
7	Known or suspected congenital infection including syphilis, cytomegalovirus, or toxoplasmosis		

If **YES** is answered to any of the above, the infant is a screen failure and will not continue in the study.

1.1. Does the Subject meet all the Inclusion Criteria and none of the Exclusion Criteria specified in the protocol? ☐ Yes ☐ No

_______________________________________ _________________________

Principal Investigator/Sub-Investigator Signature **Date**

Form 1 *(page 2)*

Patient Eligibility 2 – 24 hrs. of Age

1.2 Informed Consent -

1.2.1. Date ICF signed: _________ /_________ /_________ Goes into Medrio
dd mmm yyyy

1.2.2. Time ICF signed (24-hr clock): ____: ____

1.3. Was a waiver granted? ____ Yes ____ No

1.3.2. Date waiver granted*: ____ /______ /_____
DD MMM YYYY

1.3.3. For which I/E was the waiver granted: ________

1.3.3. Person granting waiver: _______________________

Enter information on Pre-Screening Log

1.4. Record if infant was enrolled into the study. Select Yes or No.

1.4.1. If YES, Record whether infant randomized to Active or Control

1.4.2. If NO, the infant is a **Screen Failure**. Please select the reason for screen failure:

1.4.2.1. Inclusion Criteria not met: *Specify which criteria number(s)* ________

1.4.2.2. Exclusion Criteria met: *Specify which criteria number(s)* ________

1.4.2.3. Other, Specify: ____

Completed by (signature): ___________________________ Date Completed: _______________________

Participant Number: _______________ Site Number: ______

Form 2

Randomization and Infant Demographics

Randomization

2.1. Record infant study ID from Enrollment Log:
1-__ __ __-__ __ __-1
Select "Add Patient" in Medrio and enter Study ID.

2.2. Enter infant's Birthweight Strata into Medrio to obtain randomization assignment (circle one).
____ 900 g - 1199 g or
____ 1200 g - 1999 g

2.3. Assigned Randomization Arm : ____ Active (Assignment will come from Medrio Randomization)
____ Control

2.4. Date of Randomization*: ____ /_____ /_____ *These will come from Randomization email sent from Medrio.
DD MMM YYYY

2.5. Time of Randomization*: ___ __: ___ ___

Infant Demographics

2.6. Gestational Age at Birth: _____ Weeks _____ Days *(If unknown, leave days blank)*

2.7. GA determined by (check all that apply):
___ US performed < 20 weeks
___ Ballard Score
___ Foot Length

2.8. Infant's birthdate (BD): ____ /_____ /_____
DD MMM YYYY

2.9. Time of Birth: ____: ____ (24-hour clock)

2.10. Gender: ____ Female
____ Male

Completed by (signature): ___________________________ **Date Completed: _______________________**

Participant Number: _______________ Site Number: ______

Form 3

Infant Delivery Information

3.1. Delivery Route: ____ Vaginal

____ Caesarean

3.1.1. If Cesarean, was mother in labor?

____ YES

____ NO

3.1.2. If Cesarean, indication: (Select primary indication-select one)

______ MATERNAL (Hypertension related - Pre-eclampsia/Eclampsia/HELLP)

______ MATERNAL (Previous Cesarean Section if mom in preterm labor)

______ MATERNAL (Placental: Placenta Previa, Abruptio Placenta, Antepartum Haemorrhage)

______ FETAL: (Suspicious or pathological CTG/Fetal doppler/IUGR)

______ FETAL: (Presentation - Breech or abnormal lie/presentation)

______ OTHER: Specify Other_______________________________

3.2. Delivery Resuscitation Required:

____ YES

____ NO

3.2.1. *If yes to above,* specify type of resuscitation (check all that apply):

Selections:

______ Oxygen

______ Mask CPAP

______ Positive Pressure ventilation

______ Chest Compressions

3.3. Apgar Scores:

1 Minute After Birth: ______

5 Minutes After Birth: ______

10 Minutes After Birth: ______

3.4. Temperature within first hour of admission to NICU:

___ < 36.0 C

___ 36.0 - 37.5 C

___ > 37.5 C

Completed by (signature): ___________________________ **Date Completed: _______________________**

Participant Number: _______________ Site Number: ______

Form 4 *(page 1)*

Maternal/Perinatal Data

4.1. Maternal Age (years): __________

4.2. Maternal Pregnancy History:

4.2.1. Gravida (Total number of pregnancies, including current): ______

4.2.2. Para (Total number of completed pregnancies beyond 20 weeks (whether viable or non-viable, including current): ______

4.3. Multiple gestation at this birth:

____ YES*
____ NO

(If yes to above)*

4.3.1. Type of multiple gestation: ______ MCDA
______ MCMA
______ DCDA
______ Unknown

4.4. Birth order of this infant: (Select A or B)

_____A
_____B

4.5. Was a sibling enrolled? ____ Yes
____ No

4.5.1. Sibling ID:

1 -___ ___ ___- ___ ___ ___ -1

4.6. Pre-natal Care (any):

____Yes
____No

4.6.1. Length of Pre-natal Care:

_____≥ 1 month prior to delivery
_____< 1 month prior to delivery
_____Unknown

4.7. Prenatal Steroids:

___ None
___ Optimal
(2 doses 24 hours apart, < 7 days prior to delivery but > 24 hours prior to delivery)
___ Incomplete (1 dose only OR 2nd dose received < 24 hours prior to delivery
___ Unknown

4.8. Rupture of Membranes: **choose one:**

____ ≥ 18 Hours Prior to Delivery
____ < 18 Hours Prior to Delivery
____ At delivery
____ Unknown

4.9. Chorioamnionitis: **choose one:**

____None
____Isolated Maternal Fever
____Suspected Intraamniotic Infection
____Confirmed Intraamniotic Infection

Completed by (signature): ____________________________ **Date Completed: ________________________**

Form 4 *(page 2)*

Maternal/Perinatal Data

Maternal Concomitant Medication/Therapies:

4.10. Chronic Maternal Hypertension:

____ YES, treated
____ YES, untreated
____ NO

4.10.1. Pre-Eclampsia

____ YES
____ NO

4.11. Maternal Diabetes:

____ YES
____ NO

4.11.1. If Yes, HbA1c in last known within past 3 months (%):

___ . __ (Numeric X.X from maternal medical record)
____ Unknown

4.11.2. Treatment for Maternal Diabetes:

____ Diet/No Meds
____ Oral Meds
____ Insulin

4.12. Maternal HIV Infection:

____ YES
____ NO

4.12.1. If YES, Indicate viral Risk category:

____ High Risk (maternal VL > 1000 copies)
____ Low Risk (maternal VL ≤ 1000 copies)

4.12.2. If YES, Infant PMTCT:

_____ Nevirapine (NVP) only
_____ Nevirapine (NVP) and Zidovudine (AZT)
_____ Other: Specify ___________________________

4.12.3. If YES, Infants Birth PCR:

_____ NEGATIVE
_____ POSITIVE

(If POSITIVE:)

4.12.3.1. Antiretroviral Regimen Taken?

____ YES
____ NO

4.12.3.2. If Yes, Specify Antiretroviral Regimen:

____ NVP/AZT/3TC
____ Kaletra/AZT/3TC
____ Kaletra/ABC/3TC
____ Other, Specify ________________________________

Completed by (signature): ______________________________ Date Completed: __________________________

Form 4 *(page3)*

Maternal/Perinatal Data

4.13. Maternal Syphilis:

____ YES
____ NO

4.13.1. If YES, indicate Maternal treatment:

____ COMPLETE (once weekly for 3 doses)
____ INCOMPLETE (2 doses or less)
____ NONE

4.13.2. If NONE or INCOMPLETE, Infant treatment:

____ Stat dose Benzathine (Penicillin) IMI
____ 10 days Benzyl Penicillin IVI

4.14. Maternal TB:

____ YES
____ NO

4.14.1. IF YES, Infant TB prophylaxis:

____ YES
____ NO

Completed by (signature): ____________________________ Date Completed: ________________________

Form 5 *(page 1)*

Growth - Infant Measurements

5.1. Timepoint: **Birth**

5.2. Weight: _______ grams ☐ Unknown

5.3. Head Circumference: _______. ___cm ☐ Unknown

5.4. Recumbent Length: ________ cm ☐ Unknown

5.5. Intra-uterine Growth Restriction (Select Yes or No)
_____ Yes
_____ No

5.5.1. IF Yes to Intra-uterine Growth Restriction (Choose: ___ Symmetrical, ___ Asymmetrical)

Completed by (signature): ___________________________ **Date Completed: _______________________**

Participant Number: _______________ Site Number: ______

Form 5 *(page 2)*

Growth - Infant Measurements

Subsequent Growth Parameters: Collect Weight and Head Circumference weekly to Discharge/Death.

Click "Add" in Medrio for additional timepoints.

Timepoint	5.1. Date (dd/ mmm/yyyy)	5.2. Weight (grams)	5.3. Head Circumference (__ __. __cm)
Birth + 1 week	☐ Not Done	☐ Not Done	☐ Not Done
Birth + 2 week	☐ Not Done	☐ Not Done	☐ Not Done
Birth + 3 week	☐ Not Done	☐ Not Done	☐ Not Done
Birth + 4 week	☐ Not Done	☐ Not Done	☐ Not Done
Birth + 5 week	☐ Not Done	☐ Not Done	☐ Not Done
Birth + 6 week	☐ Not Done	☐ Not Done	☐ Not Done
Birth + 7 week	☐ Not Done	☐ Not Done	☐ Not Done
Birth + 8 week	☐ Not Done	☐ Not Done	☐ Not Done
Birth + 9 week	☐ Not Done	☐ Not Done	☐ Not Done
Birth + 10 week	☐ Not Done	☐ Not Done	☐ Not Done
Birth + 11 week	☐ Not Done	☐ Not Done	☐ Not Done
Birth + 12 week	☐ Not Done	☐ Not Done	☐ Not Done
Birth + 13 week	☐ Not Done	☐ Not Done	☐ Not Done
Birth + 14 week	☐ Not Done	☐ Not Done	☐ Not Done
Birth + 15 week	☐ Not Done	☐ Not Done	☐ Not Done
Birth + 16 week	☐ Not Done	☐ Not Done	☐ Not Done
Birth + 17 week	☐ Not Done	☐ Not Done	☐ Not Done
Discharge	☐ Not Done	☐ Not Done	☐ Not Done

Participant Number: _______________ Site Number: ______

Form 6 - AeroFact Dosing – Dose 1

NEO-INSPIRe – AeroFact CALCULATION AND DISPENSING FORM

Investigational Product: SF-RI 1 (AlveoFact) Protocol Number: APC-AF-CLN-004

Infant Subject ID: 1 - _____ _____ _____ - _____ _____ _____ - _____

Dose Number: X **1** ☐ **2**

Dosing Calculation:

Formula: Birth Weight (BW) x (216mg/kg) = Total Dose (mg) Total Dose (mg) ÷ 45mg/mL = Total volume to be administered Total Volume (mL) ÷ 2.4 mL/vial = # of trays to be dispense

Birth Weight (grams)	Birth Weight (kg)	Assigned Dose	6.3. Total Dose (mg) to administer *(Enter into Medrio)*	6.4. Total Volume (mL) to administer *(Enter into Medrio)*	# of Trays SF-RI 1 Dispensed
		216 mg/kg	___.___	___.___	
Dispensed by:		Signature of Pharmacist, Doctor or Designee			Date

Dose Delivery: Dose Delivery

6.1. Dose *(check one)* Dose 1 Dose 2

6.2. Was Dose started? Yes No

6.2.1. If No, Indicate Reason (circle) ________ Selections: Adverse Event, Met Failure Criteria, Staffing issue, Device Performance issue, Protocol Deviation, Other: Specify Other_____________. If AE, AE number: ___________

6.5. Start Date of Dose*: ____/____/____ **Note: This cannot be a partial date.*

6.6. Dose Start Time (T_0) (24-hr clock): ________

6.7. Respiratory Status at **Start** of AeroFact Dose: nCPAP______cmH_2O, FiO2 ______, SpO2 ______,

6.8. End Date of Dose*: ____/____/____

6.9. Dose Stop Time (T_{END}) (24-hr clock): ________

6.10. Total Dose of AeroFact delivered: ____________ mg (XXX.X)

6.11. Total Volume of AeroFact administered: ___________ mL (X.X)

6.11.1. If AF dose delivered was less than AF Dose ordered, give reason (circle one):

1 – Adverse Event 2 - Pt met FAILURE criteria; 3 – Staffing Issue; 4 - Device Performance Issue (complete Device Performance Form); 5 – Protocol Deviation; 6 – Other, specify: __________________________________, If A/E, # _______.

6.12. Respiratory Status at **END** of AeroFact Dose: nCPAP______cmH_2O, FiO2 ______, SpO2 ______

6.13 **Controller Serial Number:** ___ ___ ___ ___ ___

6.14 **Pod Serial Number:** ___ ___ ___ ___ ___

6.15 **Drug Delivery Circuit Serial Number:** ___ ___ ___ ___ ___ ___-___ ___ ___ ___

6.16. Was AeroFact dose paused at any point (other than vial changes, check yes/no): ____ Yes ____ No

6.16.1. If paused, indicate reason: ____

6.17. 30 minutes following completion of AeroFact dose, did the infant qualify for a repeat dose of AeroFact?
Choose ____ Yes. ____ No (*6.17. Only Displays after Dose 1).*

Dose administered by (printed name) ___________________________________Initials ______ Date ___________

Participant Number: _______________ Site Number: ______

Form 6 - AeroFact Dosing – Dose 2

NEO-INSPIRe – AeroFact CALCULATION AND DISPENSING FORM

Investigational Product: SF-RI 1 (Alveofact) Protocol Number: APC-AF-CLN-004

Infant Subject ID: 1 - _____ _____ _____ - _____ _____ _____ - _____

Dose Number: ☐ 1 X 2

Dosing Calculation:
Formula: Birth Weight (BW) x (216mg/kg) = Total Dose (mg) Total Dose (mg) ÷ 45mg/mL = Total volume to be administered Total Volume (mL) ÷ 2.4 mL/vial = # of trays to be dispense

Birth Weight (grams)	Birth Weight (kg)	Assigned Dose	6.3. Total Dose (mg) to administer *(Enter into Medrio)*	6.4. Total Volume (mL) to administer *(Enter into Medrio)*	# Trays SF-RI 1 Dispensed
		216 mg/kg	___.___	___.___	
Dispensed by:		Signature of Pharmacist, Doctor or Designee			Date

Dose Delivery: Dose Delivery

6.1. Dose *(check one)* Dose 1 Dose 2

6.2. Was Dose started? Yes No

6.2.1. If No, Indicate Reason (circle) ________ Selections: Adverse Event, Met Failure Criteria, Staffing issue, Device Performance issue, Protocol Deviation, Other: Specify Other_____________. If AE, AE number: ___________

6.5. Start Date of Dose*: ____/____/____ **Note: This cannot be a partial date.*

6.6. Dose Start Time (T_0) (24-hr clock): ________

6.7. Respiratory Status at **Start** of AeroFact Dose: nCPAP______cmH_2O, FiO2 ______, SpO2 ______,

6.8. End Date of Dose*: ____/____/____

6.9. Dose Stop Time (T_{END}) (24-hr clock): ________

6.10. Total Dose of AeroFact delivered: ____________ mg (XXX.X)

6.11. Total Volume of AeroFact administered: ___________ mL (X.X)

6.11.1. If AF dose delivered was less than AF Dose ordered, give reason (circle one):

1 – Adverse Event 2 - Pt met FAILURE criteria; 3 – Staffing Issue; 4 - Device Performance Issue (complete Device Performance Form); 5 – Protocol Deviation; 6 – Other, specify: __________________________________, If A/E, # _______.

6.12. Respiratory Status at **END** of AeroFact Dose: nCPAP______cmH_2O, FiO2 ______, SpO2 ______

6.13 **Controller Serial Number:** ___ ___ ___ ___ ___

6.14 **Pod Serial Number:** ___ ___ ___ ___ ___

6.15 **Drug Delivery Circuit Serial Number:** ___ ___ ___ ___ ___ ___-___ ___ ___ ___

6.16. Was AeroFact dose paused at any point (other than vial changes, check yes/no): ____ Yes ____ No

6.16.1. If paused, indicate reason: ____

6.17. 30 minutes following completion of AeroFact dose, did the infant qualify for a repeat dose of AeroFact?
Choose ____ Yes. ____ No (*6.17. Only Displays after Dose 1).*

Dose administered by (printed name) ______________________________________Initials ______ Date ___________

Participant Number: _______________ Site Number: ______

FORM 7a *(page 1)*

Serial Subject Data ≤ 4 h After Randomization

Q 30 minutes Randomization to 4 hours

Note: Choose representative values for the time period evaluated- infant at rest. If you change to higher level of support, record the higher level of support for that time period.

Patient Status Randomization to < 4 hours									Respiratory Status Randomization to < 4 hours								
**Time Point**	**Not Done**	**Date (dd-mmm-yyyy)**	**Time (24-hr Clock)**	**HR beats/ min**	**RR breath /min**	**Severe Apnea* (Yes/No)**	**Oral Suction**	**Nasal Suction**	**Type of Support ****	**PIP (cm H2O)**	**PEEP (cm H2O)**	**CPAP (cm H2O)**	**MAP (cm H2O)**	**FiO2 (x.xx)**	**Flow Liters per min (lpm)**	**SpO2 (%)**
Rand T0	Not Done															
Randomization + 30 min	Not Done															
Randomization + 1 hr.	Not Done															
Randomization + 1.5 hr.	Not Done															
Randomization + 2 hr.	Not Done															
Randomization + 2.5 hr.	Not Done															
Randomization + 3 hr.	Not Done															
Randomization + 3.5 hr.	Not Done															
Randomization + 4 hr.	Not Done															

Participant Number: ________________ Site Number: ______

FORM 7a *(page 2 - REFERENCE)*

Serial Subject Data ≤ 4 h After Randomization

Q 30 minutes Randomization to 4 hours

Note: Choose representative values for the time period evaluated- infant at rest. If you change to higher level of support, record the higher level of support for that time period.

***Severe Apnea**:
Severe apnoeas defined as two or more apnoeas per hour requiring bag-mask ventilation, or at clinician's discretion.
Only if Occurring in the previous 30 minutes.

****Type of Support Selections:**

- HFOV
- CMV
- Biphasic CPAP
- CPAP
- HFNC
- NC (≤ 2lpm)
- Off All Support

Type of Support Selections:
** If Type of Support Selection = HFOV,*
Enter MAP and FiO2, SpO2
** If Type of Support Selection = CMV,*
Enter PIP, PEEP, MAP, FiO2, SpO2
** If Type of Support Selection = Biphasic CPAP,*
Enter PIP, PEEP, MAP, FiO2, SPO2
** If Type of Support Selection = CPAP,*
Enter CPAP, FiO2, SpO2
**If Type of Support Selection = HFNC,*
Enter LPM, FiO2, SpO2
**If Type of Support Selection = NC,*
Enter only LPM, FiO2, SpO2

Participant Number: _______________ Site Number: ______

FORM 7b *(page 1)*

Serial Respiratory Status > 4 h to 24 h After Randomization (Hourly)

Note: Choose representative values for the time period evaluated- infant at rest. If you change to higher level of support during 1 hour time period, record the higher level of support

Time Point	Not Done (circle)	Date (dd-mmm-yyyy)	Time (24-hr Clock)	Type of Support**	PIP (cm H2O)	PEEP (cm H2O)	CPAP (cm H2O)	MAP (cm H2O)	FiO2 (x.xx)	Liters Per min	SpO2 (%)
Randomization + 5 hr.	Not Done										
Randomization + 6 hr.	Not Done										
Randomization + 7 hr.	Not Done										
Randomization + 8 hr.	Not Done										
Randomization + 9 hr.	Not Done										
Randomization + 10 hr.	Not Done										
Randomization + 11 hr.	Not Done										
Randomization + 12 hr.	Not Done										

Participant Number: _______________ Site Number: ______

FORM 7b *(page 2)*

Serial Respiratory Status > 4 h to 24 h After Randomization (Hourly)

Note: Choose representative values for the time period evaluated- infant at rest. If you change to higher level of support during 1 hour time period, record the higher level of support

Time Point	Not Done (circle)	Date (dd-mmm-yyyy)	Time (24-hr Clock)	Type of Support **	PIP (cm H2O)	PEEP (cm H2O)	CPAP (cm H2O)	MAP (cm H2O)	FiO2 (x.xx)	Liters Per min	SpO2 (%)
Randomization + 13 hr.	Not Done										
Randomization + 14 hr.	Not Done										
Randomization + 15 hr.	Not Done										
Randomization + 16 hr.	Not Done										
Randomization + 17 hr.	Not Done										
Randomization + 18 hr.	Not Done										
Randomization + 19 hr.	Not Done										
Randomization + 20 hr.	Not Done										
Randomization + 21 hr.	Not Done										
Randomization + 22 hr.	Not Done										
Randomization + 23 hr.	Not Done										
Randomization + 24 hr.	Not Done										

Participant Number: _______________ Site Number: ______

FORM 7c *(page 1)*

Serial Respiratory Status > 24 h to 72 h After Randomization (Q 3 hours)

Daily entry. *Note: Choose representative values for the time period evaluated- infant at rest. If you change to higher level of support during 3-hour time period, record the higher level of support.*

Time Point	Not Done	Date* (dd-mmm-yyyy)	Time (24-hr Clock)	Type of Support**	PIP (cm H2O)	PEEP (cm H2O)	CPAP (cm H2O)	MAP (cm H2O)	FiO2 (x.xx)	NC LPM (x.xx?)	SpO2 (%)
Randomization + 27 hr.	Not Done										
Randomization + 30 hr.	Not Done										
Randomization + 33 hr.	Not Done										
Randomization + 36 hr.	Not Done										
Randomization + 39 hr.	Not Done										
Randomization + 42 hr.	Not Done										
Randomization + 45 hr.	Not Done										
Randomization + 48 hr.	Not Done										

****Type of Support Selections:**

HFOV
CMV
Biphasic CPAP
CPAP
HFNC
NC (≤ 2 lpm)
Off All Support

Type of Support Selections*:

** If Type of Support Selection = HFOV, Enter MAP and FiO2, SpO2*
** If Type of Support Selection = CMV, Enter PIP, PEEP, MAP, FiO2, SpO2*
** If Type of Support Selection = Biphasic CPAP, Enter PIP, PEEP, MAP, FiO2, SPO2*
** If Type of Support Selection = CPAP, Enter CPAP, FiO2, SpO2*
** If Type of Support Selection = HFNC, Enter LPM, FiO2, SpO2*
** If Type of Support Selection = NC, Enter LPM, FiO2, SpO2*
**If Type of Support Selection = Off All Support, no other entry required*

Participant Number: _______________ Site Number: ______

FORM 7c *(page 2)*

Serial Respiratory Status > 24 h to 72 h After Randomization (Q 3 hours)

Daily entry. *Note: Choose representative values for the time period evaluated- infant at rest. If you change to higher level of support during 3-hour time period, record the higher level of support.*

Time Point	Not Done	Date* (dd-mmm-yyyy)	Time (24-hr Clock)	Type of Support **	PIP (cm H2O)	PEEP (cm H2O)	CPAP (cm H2O)	MAP (cm H2O)	FiO2 (x.xx)	NC LPM (x.xx?)	SpO2 (%)
Randomization + 51 hr.	Not Done										
Randomization + 54 hr.	Not Done										
Randomization + 57 hr.	Not Done										
Randomization + 60 hr.	Not Done										
Randomization + 63 hr.	Not Done										
Randomization + 66 hr.	Not Done										
Randomization + 69 hr.	Not Done										
Randomization + 72 hr.	Not Done										

****Type of Support Selections:**

HFOV
CMV
Biphasic CPAP
CPAP
HFNC
NC (≤ 2 lpm)
Off All Support

Type of Support Selections*:

** If Type of Support Selection = HFOV, Enter MAP and FiO2, SpO2*
** If Type of Support Selection = CMV, Enter PIP, PEEP, MAP, FiO2, SpO2*
** If Type of Support Selection = Biphasic CPAP, Enter PIP, PEEP, MAP, FiO2, SPO2*
** If Type of Support Selection = CPAP, Enter CPAP, FiO2, SpO2*
** If Type of Support Selection = HFNC, Enter LPM, FiO2, SpO2*
** If Type of Support Selection = NC, Enter LPM, FiO2, SpO2*
**If Type of Support Selection = Off All Support, no other entry required*

Participant Number: _______________ Site Number: ______

FORM 7d *(page 1)*

Daily Serial Respiratory Status > 72 h until Discharge/Death

Daily entry. *Note: Choose representative values for the time period evaluated- infant at rest. If you change to higher level of support, record the higher level of support.*

Time Point (Age in Days)	Not Done	Date* (dd-mmm-yyyy)	Time (24-hr Clock)	Type of Support**	PIP (cm H2O)	PEEP (cm H2O)	CPAP (cm H2O)	MAP (cm H2O)	FiO2 (x.xx)	NC LPM (x.xx?)	SpO2 (%)
4											
5											
6											
7											
8											
9											
10											
11											
12											
13											
14											

****Type of Support Selections*:**
HFOV
CMV
Biphasic CPAP
CPAP
HFNC
NC (< 2 lpm)
Off All Support

Type of Support Selections*:
** If Type of Support Selection = HFOV, Enter MAP and FiO2, SpO2*
** If Type of Support Selection = CMV, Enter PIP, PEEP, MAP, FiO2, SpO2*
** If Type of Support Selection = Biphasic CPAP, Enter PIP, PEEP, MAP, FiO2, SPO2*
** If Type of Support Selection = CPAP, Enter CPAP, FiO2, SpO2*
** If Type of Support Selection = HFNC, Enter LPM, FiO2, SpO2*
** If Type of Support Selection = NC, Enter LPM, FiO2, SpO2*
**If Type of Support Selection = Off All Support, no other entry required*

Participant Number: _______________ Site Number: ______

FORM 7d *(additional logs)*

Daily Serial Respiratory Status > 72 h until Discharge/Death

Time Point (Age in Days)	Not Done	Date* (dd-mmm-yyyy)	Time (24-hr Clock)	Type of Support**	PIP (cm H2O)	PEEP (cm H2O)	CPAP (cm H2O)	MAP (cm H2O)	FiO2 (x.xx)	NC LPM (x.xx?)	SpO2 (%)

****Type of Support Selections*:**

HFOV
CMV
Biphasic CPAP
CPAP
HFNC
NC (< 2 lpm)
Off All Support

Type of Support Selections*:

** If Type of Support Selection = HFOV, Enter MAP and FiO2, SpO2*
** If Type of Support Selection = CMV, Enter PIP, PEEP, MAP, FiO2, SpO2*
** If Type of Support Selection = Biphasic CPAP, Enter PIP, PEEP, MAP, FiO2, SPO2*
** If Type of Support Selection = CPAP, Enter CPAP, FiO2, SpO2*
** If Type of Support Selection = HFNC, Enter LPM, FiO2, SpO2*
** If Type of Support Selection = NC, Enter LPM, FiO2, SpO2*
**If Type of Support Selection = Off All Support, no other entry required*

FORM 8

Treatment Failure

For Active or Control Subjects

8.1. Did the infant meet Failure Criteria within first 72 hours of life?

_____Yes
_____No

8.1.1. If Yes, Indicate Date and Time Treatment Failure Met:
Date ____ / ___ / ____ time_______
dd mmm yyyy HH:MM

8.1.2. If yes, record the respiratory parameters at the time of treatment failure:
NCPAP/PEEP _______ cm H_2O
F_iO_2 _______
S_PO_2 _______ %

8.1.3. Select all reasons for Failure that apply: **(Checkboxes)**

___ $F_iO_2 > 0.40$ on nCPAP at 5-7 cm H_2O to maintainS_PO_2 90-95% for (maintain for at least 15 min) (If Control patient on SiPaP, record PEEP pressure.

___ Severe apnoeas defined as two or more apnoeas per hour requiring bag-mask ventilation, or at clinician's discretion.

___ Severe increased work of breathing (at clinician's discretion) not responding to CPAP optimization, reposition of infant or suctioning of secretions.

___ Other Specify Other: ___________________________________

8.2. Did the infant receive bolus surfactant within 2 hours after meeting failure criteria?

____Yes If yes, complete Bolus Surfactant Instillation record.
____No

8.2.1. If no, indicate Reason: (Choose one)

____ Etiology not related to progression of RDS
____ Clinical decision to observe
____ Other; please specify____________

Treatment Failure determined by: _____________________ __________________
Signature Date: (DD-MMM-YYYY)

Completed by (signature): _________________________ **Date Completed: ___________________**

FORM 9

9.1. Intubations and Extubations Log (Form)

Was Infant intubated? Select, ____Yes or ____ No.

If No, Skip all other fields on form. If yes, complete table below:

Intubation Date/Time/Reason for Intubation							Extubation Date/Time/Rationale					
9.2. Intubation #	9.3. Date Intubated	9.4. Time Intubated	9.5. Reason for Intubation*	9.5.1. Specify Other	9.6. FiO2 (prior to Intubation) 0.21 – 1.00	9.7. Treatment Pressure CPAP/PEEP or MAP (prior to intubation)	9.8. Date Extubated	9.9. Time Extubated	9.10. Extubation Reason**	9.10.1 Specify Other	9.11. FiO2 (prior to extubation) 0.21 – 1.00	9.12. MAP Prior to Extubation

***9.5. Reason for Laryngoscopy/Intubation (choose one):**

LISA
INSURE
ETT for Surf & Vent
ETT for Resp Support
ETT for Surgery only
Other; Specify___________________________

** If Intubation Reason = LISA OR Extubation Reason = LISA, Skip – FiO2 and MAP prior to Extubation*

****9.10. Reason for Extubation (choose one):**

LISA
INSURE
Planned Extubation
Accidental Extubation
Died/Discharged on Vent**
Other; Specify_________________________________

***If Extubation Reason is recorded as Died/Discharged on Vent: Please enter the Extubation Date as Date of Death or Discharge and Extubation Time as Date of Death or Discharge*

FORM 10

Bolus Surfactant Instillation Log - Dose 1

10.1. Did Subject receive a bolus instillation of surfactant?

____ Yes
____ No

10.2. Dose Number:

____ Dose 1

____ Dose 2

____ Dose 3

10.3. How was the Bolus Instillation of Surfactant delivered?

____ LISA (Cannula/Catheter)

____ INSURE (ETT ≤ 2 hours)

____ Endotracheal Tube (> 2 hours)

10.4. Date and Time of Administration*: ____ /_____ /_____ ____: ____
DD MMM YYYY (24-Hour Clock)

10.5. Parameters immediately prior to instillation of bolus surfactant:

nCPAP /PEEP _______ cm H_2O
F_iO_2 _______
S_PO_2 _______ %

10.6. Type of bolus instilled surfactant:

___ Curosurf

___ Survanta

10.7. Volume of Instilled Dose: ______ mL (xx.x)

Did subject receive another bolus instillation of surfactant?

____Yes

____No

Completed by (signature): _______________________ **Date Completed:___________________**

FORM 10

Bolus Surfactant Instillation Log - Dose 2

10.1. Did Subject receive a 2nd bolus instillation of surfactant?

____ Yes

____ No

10.2. Dose Number:

____ Dose 1

____ Dose 2

____ Dose 3

10.3. How was the Bolus Instillation of Surfactant delivered?

____ LISA (Cannula/Catheter)

____ INSURE (ETT ≤ 2 hours)

____ Endotracheal Tube (> 2 hours)

10.4. Date and Time of Administration*: ____ /_____ /_____ ____: ____

DD MMM YYYY (24-Hour Clock)

10.5. Parameters immediately prior to instillation of bolus surfactant:

nCPAP /PEEP _______ cm H_2O

F_iO_2 _______

S_PO_2 _______ %

10.6. Type of bolus instilled surfactant:

___ Curosurf

___ Survanta

10.7. Volume of Instilled Dose: ______ mL (xx.x)

Did subject receive another bolus instillation of surfactant?

____Yes

____No

Completed by (signature): ______________________ **Date Completed:** ________________

FORM 10

Bolus Surfactant Instillation Log - Dose 3

10.1. Did Subject receive a 3rd bolus instillation of surfactant?

____ Yes
____ No

10.2. Dose Number:

____ Dose 1

____ Dose 2

____ Dose 3

10.3. How was the Bolus Instillation of Surfactant delivered?

____ LISA (Cannula/Catheter)

____ INSURE (ETT ≤ 2 hours)

____ Endotracheal Tube (> 2 hours)

10.4. Date and Time of Administration*: ____ /_____ /_____ ____: ____
DD MMM YYYY (24-Hour Clock)

10.5. Parameters immediately prior to instillation of bolus surfactant:

nCPAP /PEEP _______ cm H_2O
F_iO_2 _______
S_PO_2 _______ %

10.6. Type of bolus instilled surfactant:

___ Curosurf

___ Survanta

10.7. Volume of Instilled Dose: ______ mL (xx.x)

Did subject receive another bolus instillation of surfactant?

____Yes

____No

Completed by (signature): ________________________ **Date Completed: _________________**

FORM 11 *(page 1)*

36 Week PMA - 36 Week Respiratory and BPD Status

11.0. Date 36 Weeks PMA *: ____ /_____ /_____
DD MMM YYYY

11.1. Infant Status at 36 weeks PMA: **choose one**

______Home
______Still In Study Hospital
______Transferred to Another Facility
______ Died

11.2. Respiratory Support at 36 weeks PMA (choose one)

___ On HFOV
___ On CMV (invasive mechanical ventilation)
___ On Biphasic CPAP
___ On CPAP
___ On HFNC
___ On NC at ≤ 2 LPM
___ Off All Support (Room air, no support)
___ Unknown

11.2.1. If Unknown, reason: ______ Transferred prior to 36 wks. and unable to reach referral hospital.

______ Other (Indicate reason____________________)

Completed by (signature): ________________________ **Date Completed: __________________**

FORM 11 *(page 2)*

36 Week PMA - 36 Week Respiratory and BPD Status

11.3. If on support, Record FiO2 requirement at 36 weeks PMA: ____ 0.21

____ 0.22 – 0.29

____ ≥ 0.30

BPD Status

11.4. Does the infant have BPD? Select one:

___ Yes, Grade I

___ Yes, Grade II

___ Yes, Grade III

___ No, off all support

___ N/A, Died

___ N/A, Born ≥ 33 weeks

___ Unknown

Grading for BPD:

Grades	Invasive MV	nCPAP or HFNC > 2L/min	NC ≤ 2L/min
I		21 %	22 – 29 %
II	21 %	22 – 29 %	≥ 30 %
III	> 21 %	≥ 30 %	

Completed by (signature): ________________________ Date Completed: __________________

Form 12

Adverse Event Table

12.1. Did Subject Experience Any Adverse Events? ______ Yes
______ No

12.2. Event Name of AE	12.3 Start Date, Time	12.4 Serious? Y or N	12.5 Severity 1, 2 or 3	12.6 Relationship to Medication Unrelated or Related	12.7 Relationship to Device Unrelated or Related	12.8. Action Taken Medication	12.9 Action Taken Device	12.10 Stop Date – or continuing at Discharge	2.11 Event Outcome
	____/____/______<br>____:_____							____/____/______	
	____/____/______<br>____:_____							____/____/______	
	____/____/______<br>____:_____							____/____/______	
	____/____/______<br>____:_____							____/____/______	
	____/____/______<br>____:_____							____/____/______	

PI verification of AE Reporting: Name:__________________________ Initials__________ Date ___/___/_____

Form 12

Adverse Event Table (Reference Guide)

Selected Co-Morbidities as AEs:
- Culture proven Sepsis
- ICH
- Cystic PVL
- Pneumothorax req CT
- Pulmonary Hemorrhage
- PDA requiring treatment
- Hypotension req inotropes
- NEC
- Isolated GI Perforation
- ROP
- Other

12. 5. Severity. Indicate whether event was:

_____ Grade 1 = Mild

_____ Grade 2 = Moderate

_____ Grade 3 = Severe

12.6. Relationship to Study Medication. If "maybe" related, record "related."

_____ Related

_____ Unrelated

12.7. Relationship to Study Device as Related or Unrelated. If "maybe" related, record "related."

_____ Related

_____ Unrelated

12.8. Action Taken with Study Medication.

_____ None

_____ Dose Interrupted

12.9. Action Taken with Study Device

_____ None

_____ Removal of Controller/Delivery Circuit

_____ Not Applicable

12.10. Indicate STOP date ____/_____/_____ or

_____ Continuing at Discharge

12.11. Event Outcome

_____ Recovered/Resolved

_____ Recovering/Resolving

_____ Recovered/Resolved with Sequelae

_____ Not Recovered/Not Resolved

_____ Fatal

FORM 13

Surgeries (Form)

13.1. Were Any Surgeries Performed? **Selections:**
Yes
No

If yes, choose from VON 2022 Surgical list (MOP Appendix 5)

13.2. Organ System/Surgical Procedure	13.3. Date of Surgery	13.4. Additional Description

FORM 14

Transfusions

14.1. Were any Transfusions Performed?

____ Yes

____ No

14.2. Transfusion Type*	14.3. Volume in mL infused (numeric value only XX)	14.4. Transfusion Start Date (Dd/mmm/yyyy)

***Transfusion Types:**

Packed Red Blood Cells (PRBC)

Fresh Frozen Plasma (FFP)

Platelets

Whole Blood

FORM 15

Concomitant Medications

15.1. Did Subject take any Concomitant Medications:

____ Yes

____ No

Note: If more than one course, enter a new log line for each.

15.2. Medication*	15.2.1. Specify Other	15.3. Indication**	15.3.1. Specify Other	15.4. Start Date*** (dd/mmm/yyyy)	15.5. Stop Date*** (dd/mmm/yyyy)	15.5.1. Ongoing (checkbox)
				___/___/____	___/___/____	
				___/___/____	___/___/____	
				___/___/____	___/___/____	
				___/___/____	___/___/____	
				___/___/____	___/___/____	
				___/___/____	___/___/____	
				___/___/____	___/___/____	
				___/___/____	___/___/____	

***Medication Choices**

___ Adrenaline

___ Caffeine

___ Dexamethasone

___ Dobutamine

___ Dopamine

___ Furosemide

___ Hydrochlorothiazide

___ Hydrocortisone

___ Ibuprofen

___ Paracetamol

___ Spironolactone

___ Other, Specify___________

****Indication Choices**

___ Prevention or Treatment of Apnea

___ Prevention of BPD

___ Treatment of BPD

___ Treatment of PDA

___ Treatment of Hypotension

___ Other, Specify ________________

FORM 16

Discharge from NICU (*page 1)*

16.1. Discharge Disposition:

____ Home
____ To another hospital or facility
____ Died
____ N/A (Withdrawn prior to discharge/death)

16.1.1. If transferred to another hospital, reason:

______ Growth/discharge planning
______ Surgery
______ Diagnostic services
______ Other 16.2.1.1. Specify Other_______________________

16.2. Date of Discharge/Death *: ____ /_____ /_____
DD MMM YYYY

16.3. Respiratory status at Initial Disposition/Discharge (D/C from your NICU) (Patients not Withdrawn):

___ HFOV
___ CMV
___ Biphasic CPAP
___ CPAP
___ HFNC
___ NC ($\leq$ 2 lpm)
___ Off all support

16.4. Record FiO2 requirement at Discharge (choose one from list) (Patients not Withdrawn).

____ 0.21

____ 0.22 – 0.29

____ $\geq$ 0.30

16.5. Date off All Support ____/____/______ ____ N/A (If Discharged on Support)
DD MMM YYYY

16.6. Feedings at initial disposition

___ Breast milk only
___ Breast milk and Formula
___ Formula Only
___ N/A (not discharged to home)

Completed by (signature): ________________________ Date Completed: __________________

FORM 16

Discharge from NICU (*page 2)*

16.7. Was Consent withdrawn?

___ YES, by Parents*
___ YES, by PI**
___ NO

16.7.1. *If Parent(s) withdrew consent,

Date ___/___/___ and Time ___: ___ withdrawn from study

16.7.2.**If PI withdrew baby from study,

Date ___/___/___ and Time ___: ___ withdrawn from study

16.7.3. Reason PI withdrew Subject from study (Record reason-text field):

____

Completed by (signature): ________________________ Date Completed: _________________

FORM 17

Protocol Deviations

17.1. Did Subject have any Protocol Deviations?

____ Yes

____ No

17.2.<br>Date of Deviation<br>(dd/mmm/yyyy)	17.3.<br>Deviation*	17.4.<br>Specify Other Details (Including I/E Criterion Numbers if applicable)

***Deviation Selections:**

___ Inclusion/Exclusion criteria

___ Randomization

___ AeroFact dosing

___ Bolus instilled surfactant dosing

___ Assessments

___ Other

Completed by (signature):______________________________ **Date:_______________________**

FORM 18

Device Performance

18.1. Were there any Device Performance Issues?

____ Yes
____ No

18.2. Date of Device Performance Issue: ____ /_____ /_____
DD MMM YYYY

18.3. AeroFact Dose Number: ☐ 1 ☐ 2

18.4. Component that had the performance issue (check all that apply):

☐ Controller Specify Serial Number: __________________ Error Code: _______

☐ Drug Delivery Circuit Specify Serial Number: ___ ___ ___ ___ ___ ___ -___ ___ ___ ____

Error code (Comment) ____________________________________

☐ Pod Specify Serial Number: __________________ Error Code: _______

☐ Other Component Describe ____

18.5. Was this issue related to an Adverse Event? ☐ Yes, → 18.5.1. Specify AE Number _______

☐ No

18.6. Was another replacement component used to administer drug?

Selections:

_____ Yes
_____ No

18.6.1. If yes, indicate if another device that was used:

☐ N/A – no other component was used

☐ Controller Specify Controller Serial Number: _______________

☐ Drug Delivery Circuit Specify Serial Number: ___ ___ ___ ___ ___ ___ -___ ___ ___ ____

☐ Pod Specify Pod Serial Number: ____________________

18.7 Describe events leading up to device problem.

____

____

____

Once form is complete, email it to complaints@aerogenpharma.com within 48 hours and copy responsible CRA, then enter information into EDC.

Completed by (signature):______________________________________ **Date:**_______________________
